# Inhibition of the MAP3 kinase Tpl2 protects rodent and human *β*-cells from apoptosis and dysfunction induced by cytokines and enhances anti-inflammatory actions of exendin-4

**DOI:** 10.1038/cddis.2015.399

**Published:** 2016-01-21

**Authors:** E M Varin, A Wojtusciszyn, C Broca, D Muller, M A Ravier, F Ceppo, E Renard, J-F Tanti, S Dalle

**Affiliations:** 1 INSERM U1191, Institute of Functional Genomic (IGF), CNRS UMR5203, Montpellier University, Montpellier, France; 2 Laboratory of Cellular Therapy of Diabetes (LTCD), Institute of Regenerative Medicine and Biotherapy (IMRB), University Hospital of Montpellier, Montpellier, France; 3 Department of Endocrinology, Diabetes, and Nutrition, University Hospital of Montpellier, Montpellier, France; 4 INSERM U1065, Mediterranean Center of Molecular Medicine, University of Nice Sophia-Antipolis, Faculty of Medicine, Nice, France

## Abstract

Proinflammatory cytokines exert cytotoxic effects on *β*-cells, and are involved in the pathogenesis of type I and type II diabetes and in the drastic loss of *β*-cells following islet transplantation. Cytokines induce apoptosis and alter the function of differentiated *β*-cells. Although the MAP3 kinase tumor progression locus 2 (Tpl2) is known to integrate signals from inflammatory stimuli in macrophages, fibroblasts and adipocytes, its role in *β*-cells is unknown. We demonstrate that Tpl2 is expressed in INS-1E *β*-cells, mouse and human islets, is activated and upregulated by cytokines and mediates ERK1/2, JNK and p38 activation. Tpl2 inhibition protects *β*-cells, mouse and human islets from cytokine-induced apoptosis and preserves glucose-induced insulin secretion in mouse and human islets exposed to cytokines. Moreover, Tpl2 inhibition does not affect survival or positive effects of glucose (i.e., ERK1/2 phosphorylation and basal insulin secretion). The protection against cytokine-induced *β*-cell apoptosis is strengthened when Tpl2 inhibition is combined with the glucagon-like peptide-1 (GLP-1) analog exendin-4 in INS-1E cells. Furthermore, when combined with exendin-4, Tpl2 inhibition prevents cytokine-induced death and dysfunction of human islets. This study proposes that Tpl2 inhibitors, used either alone or combined with a GLP-1 analog, represent potential novel and effective therapeutic strategies to protect diabetic *β*-cells.

It is now clear that chronic inflammation is a hallmark of type I and type II diabetes, affecting both *β*-cell mass and insulin secretion.^1^ Type I diabetes is characterized by drastic decreases in *β*-cell mass and insulin secretion, in part mediated by proinflammatory cytokines produced following autoimmune activation.^1^ Proinflammatory cytokines, particularly interleukin-1*β* (IL-1*β*), in combination with interferon-*γ* (IFN-*γ*) and/or tumor necrosis factor-*α* (TNF-*α*), promote death by apoptosis and decrease function of differentiated *β*-cells, leading to *β*-cell destruction.^1^ Pancreatic islet transplantation is a promising alternative therapy for some type I diabetic patients.^2^ However, clinical outcome is not always achieved because of significant loss of islet mass during and after transplantation.^3^ Up to 80% of transplanted islets can die during the post-transplantation period as a result of apoptosis because of several mechanisms, notably the instant blood-mediated inflammatory response (IBMIR) and the release of a mix of cytokines including IL-1*β*, TNF-*α* and IFN-*γ*.^4^

Immune-modulatory strategies for type I diabetes therapy and improvement of islet transplantation outcomes have emerged, targeting a single specific cytokine, such as IL-1*β* or TNF-*α*.^2, 5^ However, these strategies may only target inflammation partially.^2^ Indeed, multiple cytokines, originating from surrounding immune cells and/or *β*-cells themselves, are more likely to be present simultaneously^4, 6^ and act synergistically to induce *β*-cell death and dysfunction.^7, 8, 9^ Preclinical and clinical studies demonstrated that glucagon-like peptide-1 (GLP-1) analogs, in addition to regulating glucose homeostasis *in vivo*, contribute to the restoration of normoglycemia after islet transplantation.^10, 11, 12, 13^ GLP-1 receptor (GLP-1R) analogs protect *β*-cell survival and function from proinflammatory cytokine attack.^12, 14, 15^ However, some studies have shown only modest and short-term anti-inflammatory effects of GLP-1 analogs when used alone.^11, 13, 16^

Mitogen-activated protein kinases (MAPKs) (i.e., extracellular-regulated kinase-1/2 (ERK1/2), c-Jun N-terminal kinase (JNK) and p38 MAPK) play important roles in cytokine-induced *β*-cell dysfunction and death.^1^ Conversely, ERK1/2 are involved in the beneficial effects of glucose and GLP-1 analogs.^17, 18, 19^ In this context, upstream protein kinases that specifically control the activation of MAPK in response to a combination of inflammatory cytokines (IL-1*β*, TNF-*α* and IFN-γ), rather than a single cytokine, may be useful targets for therapeutic interventions against pancreatic *β*-cell failure.

The serine/threonine kinase tumor progression locus 2 (Tpl2) (also known as COT (Cancer Osaka Thyroid) in humans) is a member of the MAP3K family (the MAP3K8) whose activation stimulates primarily the ERK1/2 pathway, but also JNK and/or p38 MAPK in some cell types, specifically in response to various inflammatory stimuli.^20, 21, 22^ Dysregulation of Tpl2 expression and signaling is associated with acute and chronic inflammatory diseases,^20, 21, 22^ and several studies highlight a critical function of Tpl2 in the control of inflammatory responses and survival in adipocytes, fibroblasts and immune and epithelial cells.^21, 22, 23, 24^

However, there is currently nothing known about the effects of Tpl2 in *β*-cells. The aim of this study was to determine whether Tpl2 may be a new key inflammatory regulator in *β*-cells or islets. We demonstrate that Tpl2 contributes to cytokine-induced *β*-cell apoptosis and dysfunction, and suggest that Tpl2 inhibition, either alone or combined with a GLP-1 receptor agonist, represents a potential new therapeutic strategy for the treatment of diabetes.

## Results

### Tpl2 is expressed and specifically activated by proinflammatory cytokines in rodent pancreatic *β*-cells lines, mouse and human islets

**Figure 1:**
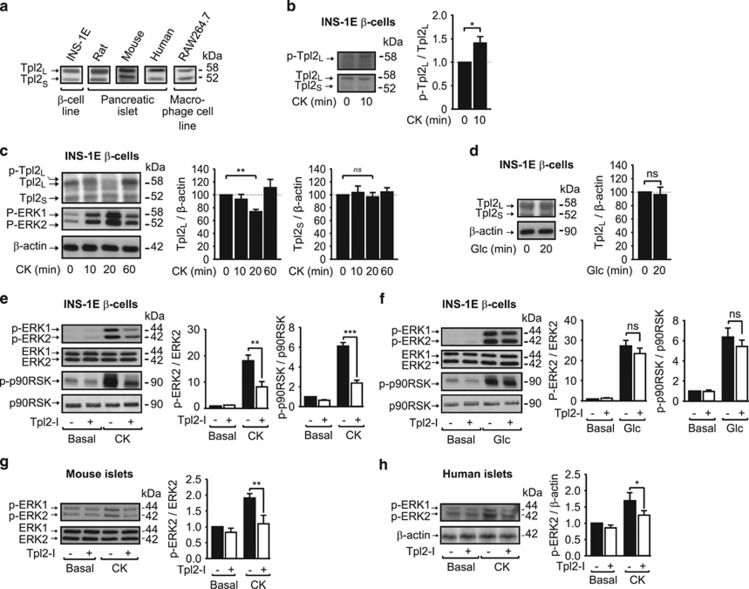
Tpl2 is expressed in *β*-cells and rodent pancreatic islets and activated by proinflammatory cytokines. Tpl2 protein expression in (**a**) INS-1E cell line, mouse, rat and human islets and macrophage RAW264.7 cell line; or in (**c**) INS-1E treated for the indicated period of time with a cytokine mix (CK); or in (**d**) INS-1E treated for 20 min with glucose (Glc, 10 mM). (**b**) Tpl2 phosphorylation (Ser400) in INS-1E stimulated for 10 min with the CK. (**e** and **f**) Western blot analysis of phosphorylated and total proteins for ERK1/2 and p90RSK in INS-1E cells, pretreated without (black bars) or with (white bars) a Tpl2 inhibitor (Tpl2-I, 3 *μ*M) for 2 h and then unstimulated (Basal) or stimulated with CK (**e**) or Glc 10 mM (**f**). (**g** and **h**) Western blot analysis of phosphorylated and total proteins for ERK1/2 in mouse islets (**g**) or human islets (**h**) treated with a Tpl2 inhibitor as in (**e**), and stimulated with CK. Representative immunoblots and quantification of three to six independent experiments are shown and expressed as a percentage of the ratio of p-Tpl2 to total Tpl2 (**b**), of Tpl2 to *β*-actin protein amount in untreated cells (**c** and **d**) or as ratio of phosphorylated to total protein amount and fold of phosphorylation over basal in cells without treatment (**e–h**). Data are presented as mean±S.E.M. *P<0.05, **P<0.01, and ***P<0.001 *versus* corresponded indicated controls (*t*-test (**b**, **d**) or one-way analysis of variance (**c**, **e**–**h**))

We first determined whether Tpl2 was expressed in INS-1E *β*-cells, and rodent and human islets. By western blot analysis, we detect two bands of 58 and 52 kDa (Figure 1a), consistent with the long (Tpl2_L_) and the short (Tpl2_S_) isoforms of Tpl2, respectively, that represent two alternative translation initiation sites described most notably in macrophages.^21^

In macrophages, adipocytes and/or fibroblasts, Tpl2 activation requires phosphorylation (i.e., Thr290, Ser400, Ser62, Ser443) by several upstream kinases including IKK*β* and Akt,^21^ and following activation, Tpl2_L_ is rapidly sequentially phosphorylated as a signal for degradation by the ubiquitin–proteasome system.^21, 25^ Here, western blot analyses revealed a slight increase in phosphorylation on Ser400 of Tpl2_L_ (Figure 1b), as well as an up-shift in Tpl2_L_ band mobility following a 10-min stimulation of INS-1E cells with a mixture of proinflammatory cytokines (CK; IL-1*β*, IFN-*γ* and TNF-*α*) (Figure 1c). This was followed by a significant decrease in protein expression of the 58 kDa form of Tpl2 (Figure 1c), whereas the 52 kDa form was not modified. This is likely indicative of both phosphorylation and degradation of Tpl2_L_ only in response to cytokines. In contrast, glucose did not modify Tpl2 protein expression (Figure 1d), suggesting that this MAP3K is specifically activated by cytokines, but not by glucose.

To further confirm the activation of Tpl2 by cytokines, we used a low concentration of a potent reversible- and ATP-competitive Tpl2 inhibitor that displays significant selectivity over other related kinases,^26^ and evaluated the phosphorylation/activation of ERK1/2, the canonical MAPK activated downstream of Tpl2,^20, 21^ and whose activation kinetics correlates with those of Tpl2, reaching a maximum after 20 min of stimulation (Figure 1c). Phosphorylation of ERK1/2 and one of its downstream targets, p90 ribosomal S6 kinase (p90RSK), induced by 20 min of cytokine exposure, was significantly decreased by ~60% following Tpl2 inhibition in INS-1E cells (Figure 1e). In contrast, glucose-induced ERK1/2 and p90RSK phosphorylation, known to play key roles in *β*-cell function and survival,^17, 18, 19^ were not affected by Tpl2 inhibition (Figure 1f).

We then assessed whether ERK1/2 activation by inflammatory cytokines was also controlled by Tpl2 in mouse and human islets. Treatment of mouse islets (Figure 1g) and human islets (Figure 1h) with a Tpl2 inhibitor markedly reduced ERK1/2 phosphorylation induced by a 20-min stimulation with cytokines.

**Figure 2:**
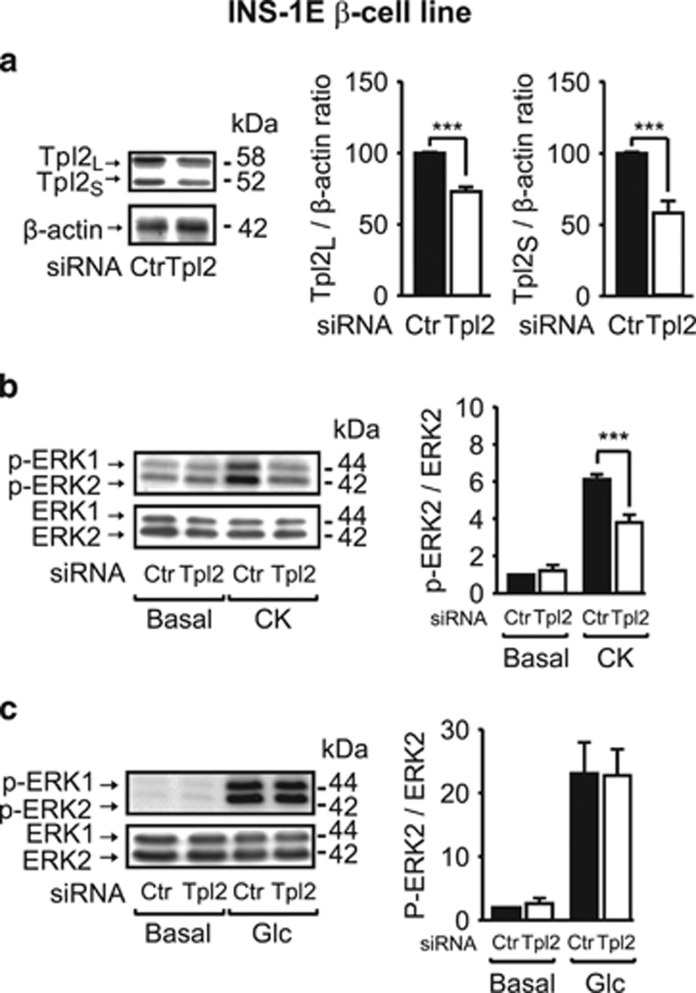
The siRNA-mediated silencing of Tpl2 in INS-1E *β*-cells decreases ERK1/2 activation induced by cytokines without affecting glucose effect. (**a**) Western blot analysis of Tpl2 expression with *β*-actin as loading control in INS-1E cells treated with control (Ctr) or Tpl2 siRNA (Tpl2). (**b** and **c**) Western blot analysis of phosphorylated and total proteins for ERK1/2 in INS-1E cells treated with Ctr or Tpl2 siRNA and incubated without (Basal) or with cytokines (CK) (**b**) or with glucose (Glc: 10 mM) (**c**) for 20 min in KRB buffer. Representative immunoblots and quantification of four to five independent experiments are shown and expressed as a percentage of the ratio of Tpl2 to *β*-actin protein amount in untreated cells (**a**), or as ratio of phosphorylated to total protein amount and fold of phosphorylation over basal in cells without treatment (**b** and **c**). Data are presented as mean±S.E.M. ***P<0.001 *versus* corresponding indicated controls (*t*-test (**a**) or one-way analysis of variance (**b** and **c**))

We further confirmed these results by using Tpl2 siRNA, achieving a 30–50% reduction in expression of both Tpl2 isoforms in INS-1E cells (Figure 2a). Under these conditions, the stimulatory effect of cytokines on ERK1/2 phosphorylation was reduced by ∼50% (Figure 2b), whereas glucose-stimulated ERK1/2 activation was not altered (Figure 2c).

### Tpl2 expression is increased by prolonged exposure of *β*-cells and human islets to inflammatory cytokines

**Figure 3:**
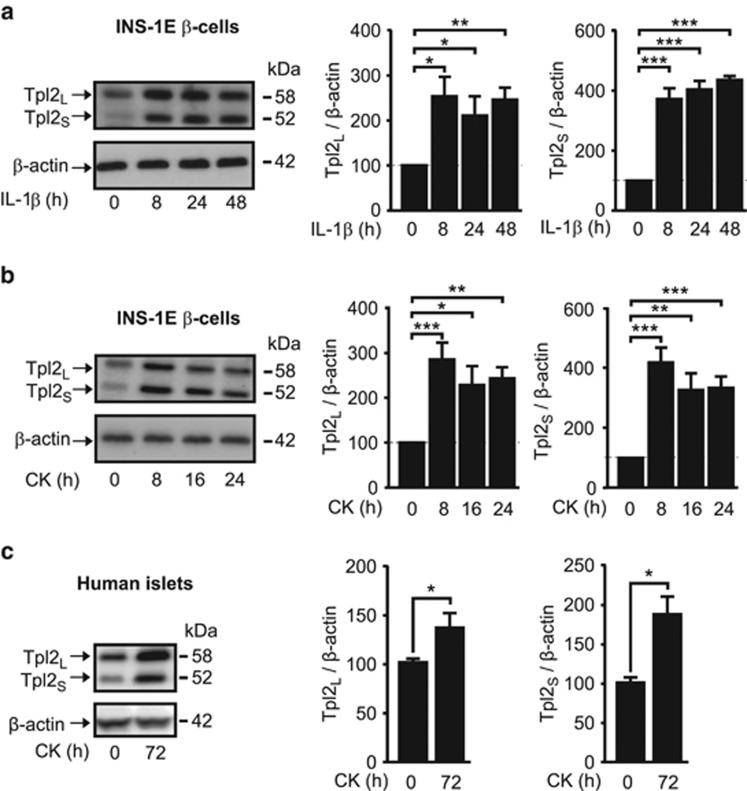
Tpl2 expression is increased by prolonged exposure of *β*-cells and human islets to inflammatory cytokines. (**a**) Tpl2 protein expression in INS-1E treated for the indicated period of time with interleukin-1*β* alone (IL-1*β*) (*n*=5). (**b**) Same as (**a**) except than cells were treated with a cytokine mix (CK) (*n*=6). (**c**) Tpl2 protein expression in human islets treated for 72 h with CK (*n*=4). Representative immunoblots and quantification of four to six independent experiments are shown and expressed as mean±S.E.M. of Tpl2 relative to *β*-actin. *P<0.05, **P<0.01 and ***P<0.001 *versus* the corresponding indicated controls (*t*-test (**c**) or one-way analysis of variance (**a**, **b**))

Tpl2 expression is positively correlated with the level of chronic inflammation in different mouse tissues and deregulates inflammatory signaling in different cell types.^21, 22^ We further examined whether prolonged treatment of INS-1E cells or isolated human islets with inflammatory cytokines modulates Tpl2 expression. Treatment of INS-1E cells with the main proinflammatory mediator IL-1*β* alone (Figure 3a), or the cytokine mixture (Figure 3b), for up to 24 h increased the expression of Tpl2_L_ and Tpl2_S_ by three- and fourfold, respectively. Importantly, Tpl2_L_ and Tpl2_S_ were also significantly increased in human islets following a 72-h exposure to the cytokine mixture (Figure 3c).

### Tpl2 inhibition protects INS-1E *β*-cells from cytokine-induced apoptosis

**Figure 4:**
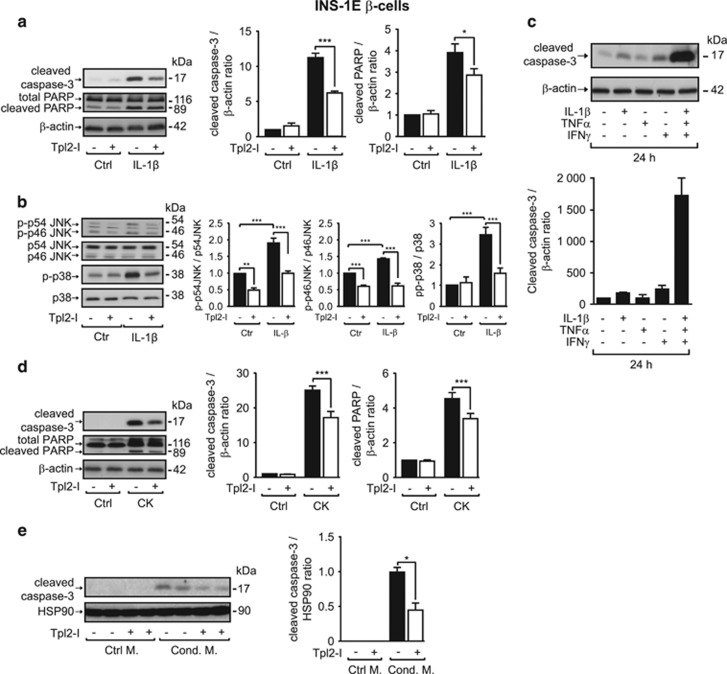
Tpl2 inhibition protects INS-1E *β*-cells from cytokine-induced apoptosis. (**a**) Level of expression of the cleaved forms of caspase-3 and PARP in INS-1E cells treated without or with Tpl2 inhibitor (Tpl2-I, 3 *μ*M) and in the absence (Control, Ctrl) or in the presence of IL-1*β* (20 ng/ml, 48 h, *n*=3). (**b**) Western blot analysis of phosphorylated and total proteins for JNK and p38 in INS-1E cells treated as in (**a**). (**c**) Level of the cleaved form of caspase-3 in INS-1E cells incubated without or with the indicated cytokines alone or in combination for 24 h. (**d**) Expression levels of the cleaved forms of caspase-3 and PARP in INS-1E cells treated without or with Tpl2 inhibitor as in (**a**) and incubated without (Ctrl) or with a cytokine mix (CK) (*n*=10). (**e**) Level of expression of the cleaved and total forms of caspase-3 in INS-1E cells pretreated without or with Tpl2 inhibitor (Tpl2-I, 5 *μ*M) for 1 h and exposed for 24 h to a control medium (Ctrl M) or a conditioned medium (Cond. M) from RAW macrophages activated with LPS (0.5 ng/ml, 24 h, *n*=3). Representative immunoblots and mean±S.E.M. of 3 to 10 independent experiments are shown. Data are expressed as ratio of cleaved caspase-3 or cleaved PARP to loading control: *β*-actin (**a**, **c** and **d**) or HSP90 (**e**) and normalized to cells without treatment. *P<0.05, **P<0.01, and ***P<0.001 *versus* the corresponding indicated control by one-way ANOVA

Tpl2 has been reported to be involved in apoptosis of renal tubular epithelial cells and intestinal myofibroblasts.^23, 24^ As previously described, exposure of INS-1E cells to IL-1*β* for 48 h led to a significant increase in cellular content of cleaved forms of caspase-3 and poly (ADP-ribose) polymerase (PARP) (Figure 4a), the two key executioners and markers of apoptosis. Both of these were significantly decreased by the inhibition of Tpl2 (45% and 30%, respectively; Figure 4a). The JNK and p38 MAPK, both involved in *β*-cell apoptosis, were activated by a 48-h exposure to IL-1*β* (Figure 4b) and, interestingly, these phosphorylation levels were significantly decreased by the inhibition of Tpl2 (Figure 4b). Consistent with previous observations, cleaved caspase-3 levels were dramatically increased by a 24-h exposure to the cytokine mixture, compared with levels induced by each cytokine alone (Figure 4c). Even under these heightened apoptotic conditions, Tpl2 inhibition decreased levels of cleaved forms of caspase-3 and PARP in *β*-cells (30% and 25%, respectively; Figure 4d).

In islets of diabetic patients, activated immune cells produce a more complex cocktail of inflammatory cytokines, contributing to *β*-cell death.^4, 27^ To determine whether Tpl2 inhibition could also protect *β*-cells from apoptosis induced by macrophage-derived cytokines, we used a conditioned medium from LPS-treated RAW264.7 macrophages, and showed that inhibition of Tpl2 in INS-1E cells decreased the level of cleaved caspase-3 by ∼55% (Figure 4e).

### Tpl2 inhibition protects mouse islets from cytokine-induced death and alteration of glucose-induced insulin secretion

**Figure 5:**
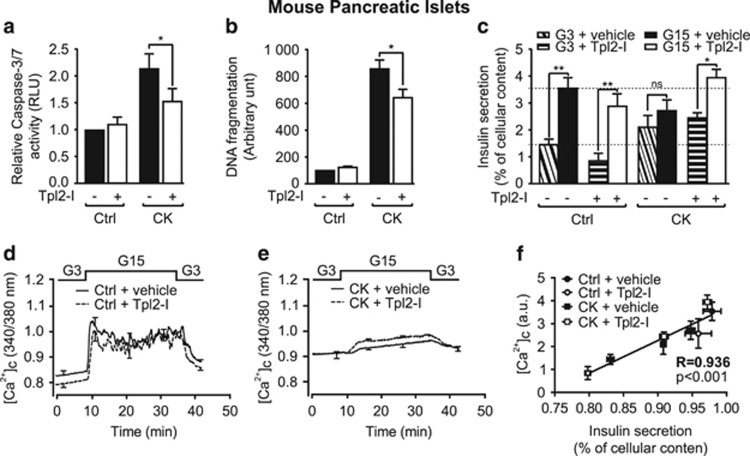
Tpl2 inhibition protects mouse islets from cytokine-induced death and alteration of glucose-induced insulin secretion. (**a**) Measurement of caspase-3/7 activity in mouse islets pretreated for 2 h without or with Tpl2-I (3 *μ*M), and then incubated for 24 h without (Control, Ctrl) or with a cytokine mix (CK). (**b**) Measurement of DNA fragmentation in mouse islets treated as in (**a**). (**c**) Glucose-stimulated insulin secretion in mouse islets treated as in (**a** and **b**) (inclined and horizontal hatched boxes: glucose 3 mM (G3), white and black solid boxes: glucose 15 mM (G15)). Each column represents mean±S.E.M. of 6 to 8 mice analyzed. *P<0.05, and **P <0.01 *versus* relative indicated controls by one-way ANOVA. (**d** and **e**) Intracellular calcium concentration ([Ca^2+^]_c_) changes monitored in perfused control islets incubated without or with 3 *μ*M of Tpl2-I (**d**) or in perfused islets treated for 24 h with the cytokine mix, and incubated without or with Tpl2-I (3 *μ*M) during 2 h before and during the CK treatment (**e**). Data are mean±S.E.M. of three experiments with three mice per group. (**f**) Correlation between insulin secretion and [Ca^2+^]_c_

We next determined whether Tpl2 was involved in the proapoptotic effects of cytokines on isolated mouse islets. We first showed that Tpl2 inhibition decreased islet cell apoptosis induced by a 24-h exposure to cytokines by ∼50% (Figure 5a), correlating with total cell death measured by DNA fragmentation (Figure 5b). As chronic exposure of *β*-cells to cytokines deteriorates not only *β*-cell survival but also function,^8, 9^ we investigated whether Tpl2 inhibition could also protect islets against the deleterious effects of cytokines on glucose-induced insulin secretion. Exposure of mouse islets to cytokines for 24 h increased basal insulin secretion, and markedly blunted the effect of glucose on insulin secretion (Figure 5c). Treatment of islets exposed to cytokines with the Tpl2 inhibitor restored glucose-stimulated insulin secretion, without modifying cytokine-induced elevated basal insulin secretion (Figure 5c). Activation of caspase-3/7 by cytokines was equivalent in islets obtained from *Tpl2*^*−/−*^ and *Tpl2*^*+/+*^ mice (Supplementary Figures S1a and b), suggesting that the upregulation of another apoptotic pathway may compensate for Tpl2 whole-body inactivation. Importantly, although Tpl2 inhibitor treatment significantly protected the islets of wild-type mice, it did not protect the islets of *Tpl2*^*−/−*^ mice from inflammatory apoptotic death (Supplementary Figures S1a and b). Moreover, cytokines had the same effect on basal and glucose-induced insulin secretion in islets from wild-type than Tpl2^−/−^ mice, but the protective effect of the Tpl2 inhibitor against altered islet function induced by the cytokines was lost in Tpl2^−/−^ islets (Supplementary Figures S1c and d). These data also indicated that the Tpl2 inhibitor elicited its effects through specific inhibition of Tpl2. Invalidation of Tpl2 did not seem to alter islet function in noninflammatory conditions as Tpl2^−/−^ mice fed a regular chow diet exhibited similar glucose tolerance and increase in insulinemia during the glucose tolerance test (Supplementary Figures S1e and f).

As insulin secretion depends primarily on calcium influx in *β*-cells, we investigated intracellular calcium concentration ([Ca^2+^]_c_) changes in mouse islets in the same conditions. Interestingly, all of the effects observed for insulin secretion in response to both cytokines and Tpl2 inhibitor were also shown for [Ca^2+^]_c_ changes (Figures 5d and e and Table 1). These two parameters strongly correlated (Figure 5f), suggesting that the Ca^2+^ increase in *β*-cells is primarily responsible for the restoration of insulin secretion.

### Combined use of Tpl2 inhibitor and exendin-4 produces a powerful anti-apoptotic effect on INS-1E *β*-cells and in human islets and protects human islets from cytokine-induced alteration of glucose-induced insulin secretion

**Figure 6:**
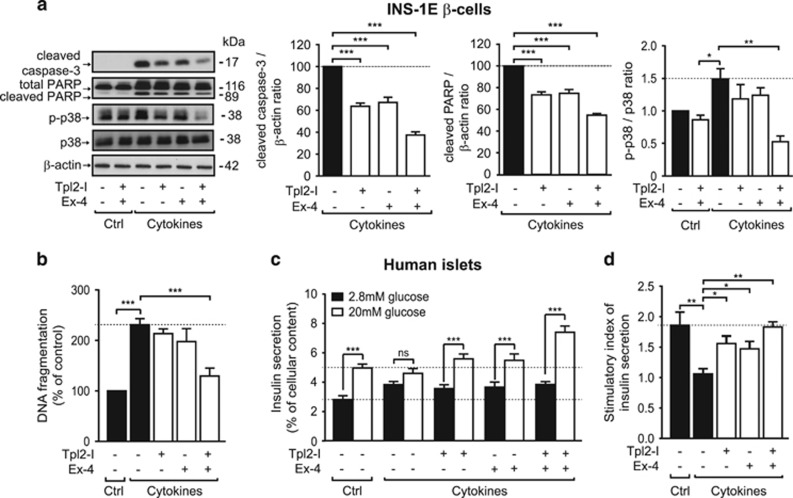
Combined use of Tpl2 inhibitor and GLP-1 analogs produces a powerful anti-apoptotic effect on INS-1E *β*-cells and in human islets and protects human islets from cytokine-induced alteration of glucose-induced insulin secretion. (**a**) Level of expression of the cleaved and total forms of caspase-3 and PARP and phosphorylated and total form of p38 in INS-1E cells treated without or with Tpl2 inhibitor (Tpl2-I, 3 *μ*M), and with or without Exendin-4 (Ex-4, 20 nM) and in the absence (Control, Ctrl) or in the presence of the cytokine mix (CK, 24 h). Representative immunoblots and mean±S.E.M. of 3 to 10 independent experiments are shown. Data are expressed as ratio of cleaved caspase-3 or cleaved PARP to loading control and normalized to cells without treatment. (**b**) Measurement of DNA fragmentation in supernatant of human islets pretreated without or with the Tpl2-I (3 *μ*M) and Exendin-4 (Ex-4, 20 nM) for 2 h and then cultured in the absence (Control, Ctrl) or in presence of the cytokine mix (CK) for 72 h in the presence or absence of Tpl2-I (3 *μ*M) and Ex-4 (20 nM). (**c**) Glucose-stimulated insulin secretion in the human islets treated as in (**b**). Black boxes represent glucose 2.8 mM (G2.8) and white boxes represent glucose 20 mM (G20). (**d**) Stimulatory index of insulin secretion from the same conditions as (**c**). Each column represents the mean±S.E.M. of 15 replicates (5 donors, each experiment in triplicate). *P<0.05, **P<0.01, and ***P<0.001 between low and high glucose (**c**) or *versus* related indicated controls (**a, b** and **d**) by one-way ANOVA

GLP-1R agonists like exendin-4 have been shown to modestly improve both islet survival and function in type I diabetic mouse models and islet transplantation studies. We evaluated whether the cytoprotective effects of activation of GLP-1R on *β*-cells could be improved by concomitant pharmacological inhibition of Tpl2. Notably, combination of pharmacological inhibition of Tpl2 and exendin-4 treatment produced a more robust anti-apoptotic effect on INS-1E cells than either agent alone (Figure 6a). Interestingly, level of p38 phosphorylation was still induced after 24 h of cytokine exposure (Figure 6a), whereas JNK and ERK phosphorylation returned to basal level or even below (data not shown). Importantly, cytokine-induced p38 phosphorylation was slightly decreased by each compound alone but was totally prevented by the combination of Tpl2 inhibitor and exendin-4.

In order to extend our results toward clinical studies, we examined whether inactivation of Tpl2 could have effects on its own and/or could improve exendin-4 protective effects on cytokine-induced death and dysfunction in human islets. Although Tpl2 inactivation and exendin-4 alone showed a trend to reduce cytokine-induced human islet cell death, the combined use of Tpl2 inhibitor and exendin-4 showed a strong protection of human islet cell viability against proinflammatory conditions (Figure 6c).

We further investigated whether this drug combination may prevent cytokine-induced insulin secretion failure. As observed in mouse islets, exposure of human islets to cytokines for 72 h severely deteriorated insulin secretion by increasing basal insulin secretion and by blocking by ~40–50% the ability of glucose to stimulate insulin secretion (Figures 6c and d). Treatment of human islets with Tpl2 inhibitor slightly but significantly restored cytokine-altered glucose-induced insulin secretion, without modifying the elevated basal insulin secretion (Figures 6c and d). Importantly, combination of Tpl2 inhibitor and exendin-4 treatment completely prevented cytokine-induced insulin secretion failure (Figures 6c and d), indicating that *β*-cells treated with this combination were viable and functional, and that human islets can be protected against the detrimental effects of cytokines.

## Discussion

We show for the first time that Tpl2 is expressed in *β*-cells, rodent and human islets and is activated in proinflammatory conditions. Importantly, Tpl2 inhibition protects INS-1E *β*-cells and pancreatic islet cells against apoptosis and dysfunction induced by proinflammatory cytokines. Notably, combination of Tpl2 inhibition and GLP-1 receptor activation confers a powerful protection of *β*-cells and human islets against cytokine injuries.

We show that both Tpl2 isoforms are expressed in *β*-cells and islets, although only the 58 kDa form of Tpl2 (Tpl2_L_) was phosphorylated and degraded. These observations, supporting an activation of Tpl2 by cytokines in *β*-cells, are in agreement with previous studies performed in immune cells,^21^ showing that Tpl2_L_ is preferentially activated and degraded.^21^ In contrast to rapid degradation following its activation, prolonged exposure to proinflammatory cytokines increased Tpl2 expression in *β*-cells. The molecular mechanism by which Tpl2 is upregulated by cytokines in *β*-cells and the final outcome of this secondary upregulation requires further investigation. As the expression of Tpl2 is upregulated by an IKK*β*/NF-*κ*B pathway in adipocytes,^22^ and as inflammatory cytokines are able to activate NF-*κ*B in *β*-cells,^1^ we can hypothesize that this pathway is involved in the enhancement of Tpl2 expression after prolonged exposure to cytokines. This ‘late' increased level of Tpl2 expression could be implicated in a reinforcement of apoptosis, as shown in other cell types^23, 24^ or in favoring signaling pathways activated by other cytokines, as proposed for adipose tissue.^22^

We demonstrated that cytokine-induced activation of Tpl2 led to ERK1/2 phosphorylation/activation. This is in agreement with what has been observed in macrophages in response to IL-1*β*^20^ or in adipocytes in response to IL-1*β* or TNF-*α*.^22^ In addition to ERK1/2, we showed that Tpl2 engaged the signaling kinases JNK and p38 in *β*-cells, as reported in microglial cells treated with LPS.^28^ This differs from MEF cells ^20, 21^ and macrophages^20^ stimulated with TNF-*α* where either only ERK1/2 and JNK or ERK1/2 and p38 are dependent on Tpl2, respectively. In *β*-cells and in rodent islets, the activation of ERK1/2, p38 MAPK, JNK and/or NF-*κ*B pathways have been proposed to play roles in cytokine-induced iNOS production, apoptosis and altered insulin secretion.^1, 29, 30, 31, 32^ Saldeen *et al.*^33^ reported that direct inhibition of ERK1/2 is not sufficient to protect against cytokine-induced apoptosis in *β*-cells, and this is in keeping with our results that demonstrate that specific inhibition of the upstream kinase Tpl2, which prevents not only ERK1/2, but also p38 and JNK signaling, protects INS-1E cells, mouse and human islets from cytokine-induced apoptosis. In addition, the inactivation of Tpl2 preserved the ability of glucose to stimulate insulin secretion in *β*-cells exposed to cytokines, indicative of maintenance of survival of insulin-secreting *β*-cells. Effects of Tpl2 inhibition on insulin secretion were likely because of restoration of [Ca^2+^]_c_ influx to a level similar to that of controls, as shown by the strong correlation between both parameters, suggesting maintenance of efficient glucose sensing and/or metabolism.

One of the strengths of our study is the potential pharmacological application of Tpl2. Indeed, Tpl2 was found to specifically control the activation of key MAPKs in response to a combination of inflammatory cytokines (IL-1*β*, TNF-*α* and IFN-γ), rather than only one of them. Inhibition of Tpl2 not only blocked the effects of IL-1*β* alone but also the detrimental apoptotic effects of a combination of IL-1*β*, IFN-*γ* and TNF-*α* as well as the apoptotic effect of cytokines secreted by LPS-activated macrophages. Hence, Tpl2 inhibition may be more efficient than anti-IL-1*β* or anti-TNF-*α* used alone, and also than NF-*κ*B blockade, that prevents *β*-cell apoptosis induced by combinations of IL-1*β* and IFN-*γ* or TNF-*α* and IFN-*γ*.^34^

The specificity of our inhibitor was supported by the absence of beneficial effects of this pharmacological tool in Tpl2^−/−^ mice on both islet survival and function. The levels of apoptosis and the alteration of insulin secretion induced by cytokines in islets from *Tpl2*^*−/−*^ and wild-type mice were similar. It can be hypothesized that even if Tpl2 plays a role in cytokine-induced *β*-cell apoptosis, in its absence from birth, other pathways may be operative and compensate for the lack of Tpl2. As an example, in macrophages isolated from whole-body Tpl2-deficient mice, JNK and p38 activation in response to LPS was sustained to compensate for the decrease of ERK1/2.^35^ Similarly, in a chemically induced mouse skin cancer, oncogenic effects of Tpl2 ablation were mediated by increased NF-*κ*B activity.^36^ Hence, inhibiting Tpl2 kinase activity, rather than inactivating its expression, may be a more efficient strategy to protect *β*-cells from proinflammatory cytokines.

Another feature of our study is that the inhibition of Tpl2 leads to protection of human islets – promoting their survival and their function – in highly inflammatory conditions. As apoptosis is more difficult to obtain using human islets, we needed to use higher concentrations and a longer exposure to cytokines. When apoptosis was induced in this model, Tpl2 inhibition alone or GLP-1R agonist alone did not significantly prevent apoptosis, and loss of function was only partially reversed by their individual use. This difference between mouse and human islets could be attributed to the lower proportion of *β*-cells in human islets as compared with mouse islets. However, these highly deleterious conditions were completely reversed by the use of Tpl2 inhibitor in combination with exendin-4. In comparison, preclinical research has demonstrated that GLP-1R agonists, such as exendin-4, protect *β*-cells from cytokine-induced apoptosis and improve islet function after islet transplantation,^10, 12, 14, 15^ but these anti-inflammatory effects were modest, especially in human islets, even if they can be potentiated by other immunomodulators.^13, 16, 37^ Our results indicate that reducing inflammatory effects by inhibiting Tpl2 strongly improves the ability of GLP-1 analogs to promote human islet survival and function.

How Tpl2 inhibition and activation of GLP-1 pathways interact remain to be fully explored but different mechanisms may underlie these effects. Our results implicate a potential role for p38, in accordance with a previous study showing that exendin-4 mediates cytoprotective effects on *β*-cells by decreasing activation of p38.^38^ Another possibility is that Tpl2 inhibition blocks an inhibitory effect of inflammatory cytokines on GLP-1 and/or other beneficial signaling pathways. In this context, it has been reported that IL-1*β*, through NO production and NF-*κ*B activation, regulates positively the expression of the inducible cyclooxygenase COX-2, its product PGE2 and its receptor EP3 in *β*-cells.^39^ Inhibition of COX-2 and EP3 expression improves *β*-cell function and prevents type I diabetes development in a mouse model.^40, 41^ Moreover, by binding to its G*α*i couple receptor EP3, PGE2 reduces cAMP concentration, antagonizes the effects of GLP-1 analogs and decreases glucose-induced insulin secretion.^39^ Interestingly, in macrophages and adipocytes, the Tpl2-ERK1/2 pathway regulates the expression of COX-2 and PGE2 secretion in response to LPS or other cytokines.^42, 43^ Thus, Tpl2 inhibition in *β*-cells may decrease the production and secretion of PGE2 induced by cytokines, and then may favor effects of GLP-1R agonists on survival and function of islets. In addition, as islets also contain infiltrated macrophages that express Tpl2 and secrete cytokines, Tpl2 inhibition in islets can also target macrophages, key players in *β*-cell failure. This highlights the potential of this pharmacological tool in diabetes by targeting both *β*-cells and macrophages.

Altogether, our study provides new insights into the molecular mechanisms involved in *β*-cell and human islet failure induced by cytokines. Moreover, our findings suggest potential new strategies clinically applicable to prevent IBMIR and increase success rates in islet transplantation, as well as for the treatment of diabetes based on Tpl2 inhibitors, alone or combined with GLP-1 analogs. The latter appears as a more attractive and efficient therapeutic strategy. Moreover, chronic inflammation is now also considered a hallmark of type II diabetes and exposure of human islets to metabolic stresses increases levels of IL-1*β* and chemokines that may contribute to insulin secretory failure and *β*-cell death in type II diabetes.^44^ Thus, our data suggest that Tpl2 inhibition may also be a relevant therapeutic strategy to preserve *β*-cell function and/or mass in type II diabetes. As Tpl2 inhibitors are currently developed by the pharmaceutical industry to treat inflammatory diseases,^45, 46^ and GLP-1R agonists are approved for treatment of type II diabetes and obesity, our results reinforce the need to first expand the preclinical evaluation of these drugs in animal models of type I and type II diabetes, and then to build new clinical evaluation in patients suffering from diabetes and/or related metabolic disorders.

## Materials and Methods

### Animals

Male C57BL/6J mice were from Charles River Laboratories (St. Aubin les Elbeuf, France). C57BL/6J WT and C57BL/6J *Tpl2*^*−/−*^ littermates were produced as described previously.^47^ All animals were maintained on a 12 h light/dark cycle and were provided free access to water and standard rodent diet (4% fat). Principles of laboratory animal care (NIH publication no. 85–23, revised 1985), and European Union guidelines on animal laboratory care were followed. All animal studies were approved by the Ministry of Agriculture, France (D34-172-13 and NCE/2012-89) and the Animal Care Committee of Nice Sophia Antipolis and Montpellier Universities.

### Islet isolation and culture of INS-1E cells and islets

The rat *β*-cell line INS-1E was provided by P Maechler (Cell Physiology and Metabolism Department, University of Geneva, Geneva, Switzerland), and cultured as previously described.^48^ RAW264.7 macrophages were cultured as previously described.^42^ Islets were isolated from WT and *Tpl2*^*−/−*^ C57BL/6J mice following the injection of 2 ml of collagenase XI (1 mg/ml) through the bile duct. Pancreases were digested for 9 min at 37 °C, and islets were isolated using a histopaque-1077 gradient. Islets were washed in cold PBS, handpicked under a microscope, separated into groups composed of 200–300 islets, and maintained in culture at 37 °C in RPMI-1640 supplemented with 10% FBS, 2 mmol/l glutamine, 100 units/ml penicillin and 100 *μ*g/ml streptomycin for 24 h. Human pancreases were harvested from five brain-dead nonobese nondiabetic donors. Experiments were performed in agreement with the Institutional Ethical Committee of the French Agence de la Biomédecine (ref. PFS10-001). Human islets were isolated at the Diabetes Cellular Therapy Laboratory (Institute for Research in Biotherapy, Montpellier, France) or at the Cell Isolation and Transplantation Center (University of Geneva, Geneva, Switzerland) according to a slightly modified version of the automated method.^49^ Human islets were cultured for recovery for 1 to 5 days after isolation in CMRL 1066 (Mediatech, Herndon, VA, USA) medium containing 5.6 mmol/l of glucose and supplemented with 10% FBS, 100 UI/ml penicillin, 100 mg/ml streptomycin and 2 mM glutamine.

### Drug exposure

Human recombinant IL-1*β*, TNF-*α*, human or murine recombinant IFN-γ were from Invitrogen (Life Technologies SAS, Courtaboeuf, France), murine IL-1*β* and TNF-*α* from PreProtech (Neuilly, France), Tpl2 kinase inhibitor from Calbiochem (Merck Millipore, Darmstadt, Germany) and Exendin-4 from Bachem (Bubendorf, Switzerland). Acute experiments (20 min) were performed in Krebs-Ringer Bicarbonate (KRB) buffer (see compositions in Broca *et al.*^48^). INS-1E cells (~70% confluence in 6-well plates), mouse islets (200–300 per condition in 1.5 ml tubes) or human islets (500–1000 IEQ per condition in 12-well plates) were preincubated at 37 °C for 2 h with or without Tpl2 inhibitor (3 *μ*M) and then incubated for the indicated times with or without Tpl2-inhibitor (3 *μ*M) in the absence (basal) or presence of glucose (10 mmol/l), IL-1*β* alone (10 000 U/ml, 20 ng/ml) or a cytokine mix (100 U/ml IL-1*β* (0. 2 ng/ml), 500 U/ml TNF-*α* (50 ng/ml) and 100 U/ml IFN-*γ* (30 ng/ml)), as indicated in the figure legends. For human islets, the cytokine mix was 1000 U/ml IL-1*β* (2 ng/ml), 1000 U/ml TNF-*α* (28 ng/ml) and 1000 U/ml IFN-*γ* (833 ng/ml).

Long-term experiments (24–72 h) were performed in RPMI-1640 medium containing 2 mM glutamine, 100 U/ml penicillin, 100 μg/ml streptomycin and glucose (11.1 mmol/l for INS-1E cells and mouse islets, and 5.6 mmol/l for human islets). The medium was supplemented with FBS (10% for INS-1E cells and 10% for mouse islets) or albumin (0.5% of BSA for IL-1*β* alone on INS-1E cells, and 1% of human albumin for human islets). INS-1E cells (~70% confluence in 6-well plates), mouse (5–10 islets per condition in 24-well plates) or human islets (500–2000 IEQ per condition in 60 mm Petri dishes) were preincubated at 37 °C for 2 h with or without Tpl2 inhibitor (3 *μ*M), and then incubated for the indicated times, with or without Tpl2 inhibitor (3 *μ*M), and in the absence or presence of IL-1*β* alone and/or a cytokine mix (same concentrations as for short-term experiments), as indicated in the figure legends. All experiments on human islets were performed at the Diabetes Cellular Therapy Laboratory of Montpellier.

RAW macrophages (5 × 10^6^ cells per 100 mm) were incubated for 24 h in 6 ml of culture medium containing LPS (0.5 ng/ml). After 24 h, the conditioned medium (CM) was collected and transferred onto INS-1E cells (0.7 ml of CM/well of a 12-well plate) treated with or without the Tpl2 inhibitor (5 *μ*M) for 1 h. For control conditions, cultured medium (Control medium) containing the same concentration of LPS and Tpl2 inhibitor was added onto the INS-1E cells. After 24 h, cells were lysed for cleaved caspase-3 analysis by western blot.

### RNA interference

In RNA interference experiments, Tpl2 expression was specifically silenced in INS-1E cells using a validated set of 4 different 19-nucleotide siRNA duplexes (‘ON-TARGETplus SMARTpool', L-091828-01-0005) purchased from Dharmacon (GE Healthcare Dharmacon Inc., Lafayette, CO, USA). Briefly, groups of 500 000 INS-1E cells were maintained in culture in the absence of penicillin and streptomycin for 24 h before being transfected with 40 nM siRNA-Lipofectamine 2000 complexes prepared in Opti-MEM medium in a 2 : 1 ratio. At 6 h after transfection, the medium was replaced with fresh antibiotic-free RPMI medium supplemented with 7.5% FCS. A second transfection was performed 24 h after the first one to improve the transfection efficiency. All assays were performed at least 50 h after the first transfection.

### Insulin secretion and [Ca^2+^]_c_ measurements

After the incubation periods, islets (5 mouse islets or 3 × 50 human IEQ per condition) were preincubated for 1 h for stabilization in KRB buffer^48^ containing glucose 2.8–3 mmol/l, followed by a 1-h incubation at 2.8–3 mmol/l and an additional 1 h at glucose 20 mmol/l (human) or 15 mmol/l (mouse). Aliquots from the incubation buffers were collected and cleared by centrifugation. Islet insulin content extraction was performed using acid ethanol, followed by sonication. Insulin release and content were measured by radioimmunoassay (Merck-Milipore, Molsheim, SAS FRANCE), and normalized to insulin content. For [Ca^2+^]_c_ measurements, cultured islets were loaded with 2 *μ*mol/l Fura2-Leak-Resistant-AM (Tef Labs, Austin, TX, USA) for 2 h, before being imaged as previously described.^50^

### Western blotting and measurements of cell and islet apoptosis

After treatments, INS-1E cells (~70% confluence in 6-well plates), mouse islets (200–300 per condition) or human islets (500–2000 IEQ per condition) were lysed as previously described,^48^ and western blot analyses were performed as previously described^17^ using the antibodies listed in Supplementary Table S1. INS-1E apoptosis was investigated by the quantification of cleaved forms of caspase-3 and PARP by western blotting. Mouse islet apoptosis was assessed by caspase-3/7 activity measurement using the Caspase-Glo 3/7 Assay (Promega Corp., Madison, WI, USA). Briefly, caspase-3/7-Glo reagent was added after 24 h incubation of islets in 96-well plates (10 islets per well), and the samples were incubated at 37 °C for 2 h. Luminescence was measured using a TECAN infinite 200 plate reader (TECAN, Männedorf, Switzerland). Total cell death in mouse and human islets was evaluated using the Cell Death Detection ELISA kit (Roche, Meylan, France) using 20 *μ*l of culture supernatant. As a consequence of our prolonged treatments, DNA fragments were present in the supernatant of cultured cells as the result of the lysis of late apoptotic cells as well as necrotic cells. Absorbance was measured at 405 nm using the Mithras LB940 Reader (Berthold, Thoiry, France) and the results expressed in arbitrary units of oligonucleosome-associated histone (DNA fragmentation).

### Statistical analysis

Results are expressed as means±S.E.M. for *n* independent experiments. The statistical significance between means was assessed by unpaired Student's *t*-test, or by ANOVA followed by Newman–Keuls *post hoc* analyses. A *P-*value of <0.05 was considered significant.

## Figures and Tables

**Table 1:** Mouse islets perfused with Tpl2-I are protected against the cytokine-induced loss of intracellular calcium concentration ([Ca^2+^]_c_) changes in response to glucose

	**Control**		**CK**	
**Tpl2-I**	**−**	**+**	**−**	**+**
Frequency (min^−1^)	0.467±0.054	0.500±0.065^a^	No	No
Response to glucose	22/22 (100%)	18/18 (100%)	7/17 (41.2%)	20/23 (87%)

*Mean* [*Ca*^*2+*^]_*c*_ *level (a.u.)*				
3 mM glucose	0.831±0.005	0.798±0.004^b^	0.908±0.004^b^	0.909±0.006^b^
15 mM glucose	0.972±0.013	0.959±0.019^a^	0.948±0.006^c^	0.972±0.006^a^^,^^d^
** **Delta response	0.142±0.013	0.161±0.020^a^	0.040±0.005^b^	0.064±0.006^b^^,^^d^

a.u., arbitrary unit, Tpl2-I, Tpl2 inhibitor, CK, cytokines

a NS compared with Control without Tpl2-I

b *P*<0.001 compared with Control without Tpl2-I

c *P*<0.05 compared with Control without Tpl2-I

d *P*<0.01 compared with Cytokines without Tpl2-I

## Abbreviations

ERK1/2: extracellular-regulated kinase-1/2
GLP-1: glucagon-like peptide-1
IBMIR: instant blood-mediated inflammatory response
IFN-*γ*: interferon-*γ*
IL-1*β*: interleukin-1*β*
JNK: c-Jun N-terminal kinase
MAPK: mitogen-activated protein kinase
PARP: poly(ADP-ribose) polymerase
TNF-*α*: tumor necrosis factor-*α*
Tpl2: tumor progression locus 2
