# Supplementary material for: Inhibition of the MAP3 kinase Tpl2 protects rodent and human β-cells from apoptosis and dysfunction induced by cytokines and enhances anti-inflammatory actions of exendin-4
Source: Cell Death Dis. 2016 Jan 21;7(1):e2065–. doi: 10.1038/cddis.2015.399 (PMC4816180; doi:10.1038/cddis.2015.399)
Supplement: Supplementary Figure 1 [file cddis2015399x1.pdf]

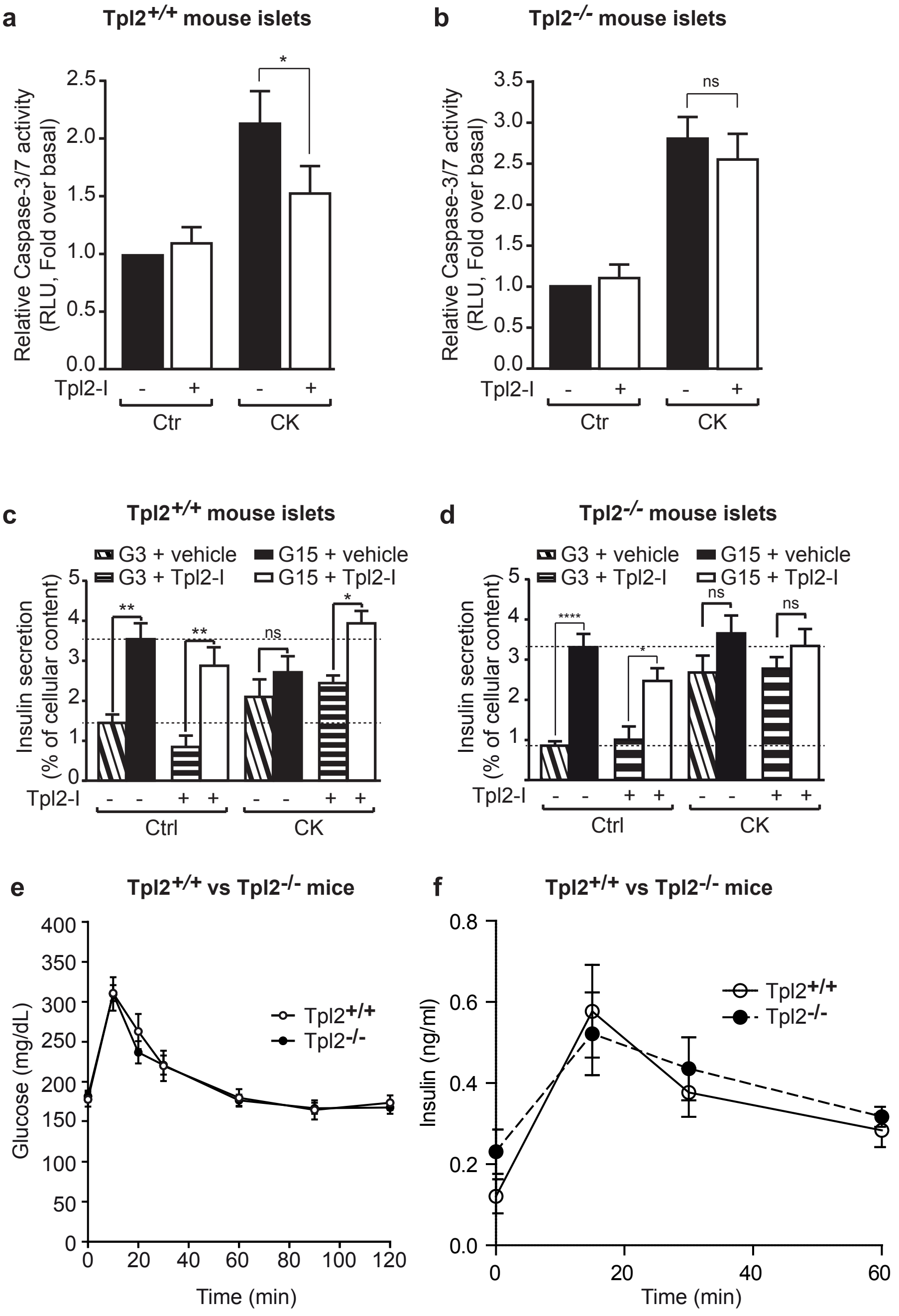

Supplementary Figure 1. Tpl2 inhibitor specifically blocks Tpl2 activity effects and Tpl2$^{-/-}$ have no improvement of survival nor function. a-b: Caspase-3/7 activity in mouse islets from **a***:* WT (Tpl2$^{+/+}$) or **b***:* Tpl2-deficient (Tpl2$^{-/-}$) untreated (Ctr) or treated for 24 h with cytokine mix (CK) with or without Tpl2-I (3 μM). Data are mean ± SEM (N=8/group). *P<0.05 vs CK (one-way ANOVA). **c-d:** Insulin secretion in response to glucose (3mmol/L (G3) vs 15mmol/L (G15)) on the same conditions that **a and b.** Data are mean ± SEM (N=8/group). *P<0.05 G15 vs G3 (one-way ANOVA). **e and f:** intraperitoneal *(ip)* Gluocse tolerance test performed in Tpl2$^{+/+}$ and Tpl2$^{-/-}$ mice (**e**), with associated plasma insulin level (**f**).
