# Supplementary material for: Inhibition of the MAP3 kinase Tpl2 protects rodent and human β-cells from apoptosis and dysfunction induced by cytokines and enhances anti-inflammatory actions of exendin-4
Source: Cell Death Dis. 2016 Jan 21;7(1):e2065–. doi: 10.1038/cddis.2015.399 (PMC4816180; doi:10.1038/cddis.2015.399)
Supplement: Supplementary Table 1 [file cddis2015399x2.pdf]

Online supplemental Table 1. List and references of antibodies

Antibody	Reference	Company
β-actin	A-5441	Sigma
Cleaved and total PARP	9542	Cell Signaling
Cleaved Caspase-3	9661	Cell Signaling
HRP-linked anti-mouse IgG	SC-2005	Santa Cruz
HRP-linked anti-rabbit IgG	7074	Cell Signaling
HSP90	05-594	Upstate Biotechnology
Phospho-p38 MAPK (Thr180/Tyr182)	4511	Cell Signaling
Phospho-p90rsk (Thr573)	9346	Cell Signaling
Phospho-SAPK/JNK (Thr183/Tyr185)	9251	Cell Signaling
Phsopho-ERK1/2 (Thr202/Tyr204)	9106	Cell Signaling
Phospho-Tpl2 (Ser400)	4491	Cell Signaling
Total ERK1/2 (p42/p44 MAPK)	610030	Transduction Laboratories
Total p38 MAPK	9212	Cell Signaling
Total p90rsk	9355	Cell Signaling
Total SAPK/JNK (p46/p54 SAPK)	9252	Cell Signaling
Total Tpl2 (C-term region)	SC-720	Santa Cruz
